# Effect of Extracorporeal Shock Wave Treatment on Deep Partial-Thickness Burn Injury in Rats: A Pilot Study

**DOI:** 10.1155/2014/495967

**Published:** 2014-11-06

**Authors:** Gabriel Djedovic, Florian Stefan Kamelger, Johannes Jeschke, Hildegunde Piza-Katzer

**Affiliations:** ^1^Department of Plastic, Reconstructive and Aesthetic Surgery, Innsbruck Medical University, Anichstraße 35, 6020 Innsbruck, Austria; ^2^Department of Traumatology and Sports Medicine, Innsbruck Medical University, Anichstraße 35, 6020 Innsbruck, Austria

## Abstract

Extracorporeal shock wave therapy (ESWT) enhances tissue vascularization and neoangiogenesis. Recent animal studies showed improved soft tissue regeneration using ESWT. In most cases, deep partial-thickness burns require skin grafting; the outcome is often unsatisfactory in function and aesthetic appearance. The aim of this study was to demonstrate the effect of ESWT on skin regeneration after deep partial-thickness burns. Under general anesthesia, two standardized deep partial-thickness burns were induced on the back of 30 male Wistar rats. Immediately after the burn, ESWT was given to rats of group 1 (*N* = 15), but not to group 2 (*N* = 15). On days 5, 10, and 15, five rats of each group were analyzed. Reepithelialization rate was defined, perfusion units were measured, and histological analysis was performed. Digital photography was used for visual documentation. A wound score system was used. ESWT enhanced the percentage of wound closure in group 1 as compared to group 2 (*P* < 0.05). The reepithelialization rate was improved significantly on day 15 (*P* < 0.05). The wound score showed a significant increase in the ESWT group. ESWT improves skin regeneration of deep partial-thickness burns in rats. It may be a suitable and cost effective treatment alternative in this type of burn wounds in the future.

## 1. Introduction

Burn wounds are a response to thermic, chemical, or electric forces and, depending on the depth of the burn, acute surgical debridement of the burned skin layers may be necessary. Wounds that affect the superficial skin layers including the reticular stratum characterize deep partial-thickness burns. Thus, destruction of structures that play an important role in skin regeneration leads to scar formation as well as to decreased functional and aesthetical outcome. However, in contrast to full-thickness burn wounds, blood supply to the affected area is still intact.

Extracorporeal shock wave therapy (ESWT) has been used for over 30 years in lithotripsy. Besides its mechanical effects, its biological effects were first described nearly 15 years ago by orthopedic surgeons. Still, the exact mechanisms are not perfectly known yet; however, experimental studies could reveal the potential of shock waves to increase growth factors, which are known to be crucial for wound healing and angiogenesis [1]. Angiogenesis is an essential component of wound healing and, thus, of healing of burn wounds. Other studies showed an increase in skin flap survival in rats or described effects improving the clinical outcome in patients with plantar fasciitis, tennis elbow, or nonunions [2–6].

The purpose of this study was to investigate the effect of ESWT on wound healing in deep partial-thickness burns and to develop a standardized protocol for an animal model that allows further investigations of burns of this kind.

Thereto, we modified the burn animal model originally described by Kaufman et al. and modified by Kuroda et al. [7, 8]. Macroscopical and histological examinations were carried out for measuring wound surface and counting epithelial layers. Additionally, a score system was used that combines epithelialization, granulation, cell density, and vascularization [9, 10] to measure the improvement in wound healing with and without ESWT.

We hypothesize that ESWT of deep partial thickness burns leads to an increase of perfusion in the wound and thus to a better healing in terms of a faster reepithelialization as well as of a lack of scar formation.

## 2. Material and Methods

### 2.1. Animals

The animals (300–380 grams) used in the present study were maintained and used in conformity with Austrian national regulations and international guidelines of the Council of Europe on animal welfare. All 30 Wistar rats (Charles River Laboratories, Sulzfeld, Germany) were kept in separate cages with a 12-hour light/12-hour night circle and free access to pellets and water. Rats were sedated with sevoflurane and anesthetized with ketamine hydrochloride. Postoperatively, the animals were treated with 0.1 mg/kg BW/12 h buprenorphine for adequate analgesia.

### 2.2. Burn Stamp

After anesthesia, the dorsal fur of the rats was trimmed and the skin depilated with Veet, a commercially available depilation cream. Anatomical structures that defined the burn areas were the iliac crest, the 12th rip, and the spinous process. In accordance with the protocol of Kaufman et al. [7], modified by Kuroda et al. [8], two standardized deep partial-thickness burns were inflicted on the dorsal skin of each animal with a specially constructed burn stamp made of steel with a tare weight of exactly 500 grams and a diameter of 2 cm. The burn stamp was preoperatively heated in a 70°C water bath for two hours. Induction of a burn wound with the heated stamp was for 9 seconds and no additional pressure was applied in order to keep standardized conditions. Preliminary histological investigation established the presence of a deep partial-thickness burn wound using the described protocol (data not shown).

### 2.3. Extracorporeal Shock Wave Therapy

After standardized infliction of two burn wounds in all animals, rats were randomly divided into two groups of 15 animals each. Group 1 was treated with ESWT, whereas group 2 served as control.

In group 1, immediately after infliction of the burn wound, the shock wave applicator (Evotron, High Medical Technologies, Lengwil, Switzerland) was held perpendicularly to the wound and 500 shocks were applied with an energy flux density of 0.11 mJ/mm^2^ and a frequency of 240/min. A commercially available ultrasonic gel served as contact medium. The burn wounds of group 2 were covered with ultrasound gel, which was then removed after two minutes. To authors' knowledge, commercially available ultrasonic gel does not influence water loss and wound healing.

The wound was not subjected to any further manipulation.

### 2.4. Analysis

Two investigators performed analysis in a blinded fashion. All wounds were photo-documented daily, pictures were transferred to hard disk, and wound surfaces were quantified with standard analysis software. Each group was divided into three subgroups. Five animals of each group were killed on day five, day ten, and day fifteen, respectively, with an overdose of pentobarbital. Before the animals were sacrificed, wounds were investigated with a Laser Doppler Imager (LDI, Moor Inc., Sussex, England) and the data were transferred to hard disk. We attached great importance to a sufficient sedation status of the animals to avoid artifacts and corruptions because of movement during LDI-measurement. Nevertheless, measurements were repeated if artifacts occurred.

Percentage of open wound surface on day_*x*_ was calculated:

(1) Ø  of  wound  surface  of  dayx  Ø  of  wound  surface  of  day1×100  =percentage  %  of  surface  on⁡  dayx.

Additionally, on day 1 and on day_*x*_, the burn wounds were measured with the LDI. Regions of interest (ROI) were defined 2 × 2 cm. Percentage of perfusion units (PU) of day_*x*_ was calculated as follows:

(2) mean  of  PU  of  dayx  mean  of  PU  of  day1×100  =percentage  %of  PU  on⁡  dayx.

Wound areas were harvested, fixed in 4% formaldehyde and embedded in paraffin wax, and prepared for histological analysis. H&E staining was performed. The number of epithelial cell layers on days 5, 10, and 15 was counted. Furthermore, wound healing was assessed histologically within the wound center with the score system of Yu et al. [9] as modified by Schlager et al. [10]; this system combines the parameters of the degree of reepithelialization, cell number, granulation tissue, and vascularization for classifying burn wounds (Table 1). The determined scores for each parameter were summed up and presented for days 5, 10, and 15.

### 2.5. Statistical Analysis

The Mann-Whitney *U* test was used. Results are expressed as mean ± standard deviation (SD) and considered significant when *P* < 0.05 and highly significant when *P* < 0.01. All statistical analyses were performed using SPSS 12.0 software (SPSS, Chicago, Illinois).

## 3. Results

### 3.1. Macroscopical Analysis

None of the animals showed any signs of infection. Animals lost 10 to 20 grams of body weight from day 0 to day 1. Body weight normalized during the study period.

**Figure 1:**
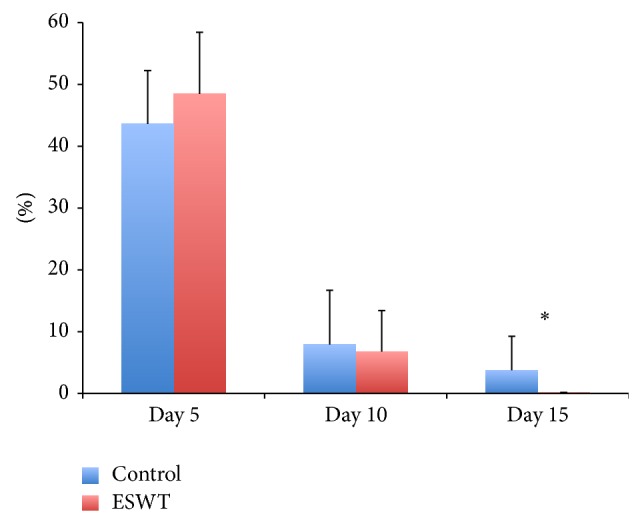
Percentage of remaining wound areas of the control group compared to the ESWT-treated group in correlation to the measurements taken on day one. Day 15 showed statistically different wound surfaces (^*^ *P* = 0.028).

Wound measurements on day 5 showed a remaining surface of 48.5% ± 10.0 in the ESWT group (group 1) and of 43.6% ± 8.6 in animals of the control group (group 2) in comparison to the measured wound area on day 1. No significance could be calculated. Day 10 showed 6.8% ± 6.6 of remaining wound surface in the ESWT group and 7.9% ± 8.8 in the control group. On day 15, a statistically significant difference between the ESWT group (0.1% ± 0.1) and the control group (3.8% ± 5.5) could be seen (Figure 1; *P* < 0.05).

**Figure 2:**
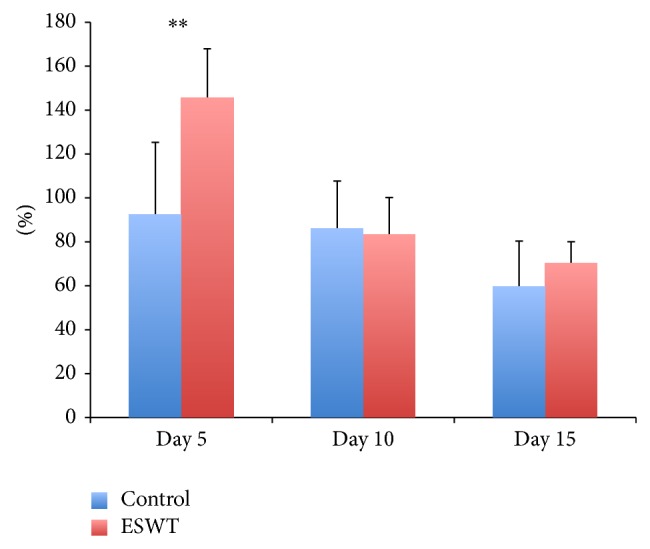
Percentage of perfusion units of the control compared to the ESWT-treated group in correlation to the measurements taken on day one. Day 5 showed statistically higher perfusion of the wound (^**^ *P* < 0,01).

Perfusion measurements using the Laser Doppler Imager showed no statistically significant difference between the two groups on day 10 (group 1: 83.3% ± 16.9, group 2: 86.0% ± 21.7) and day 15 (group 1: 70.3% ± 9.8, group 2: 59.5% ± 20.9) in comparison to the measured perfusion units on day 1. Only the measurements on day 5 showed a high statistical significance (group 1: 145.2% ± 22.7, group 2: 92.7% ± 32.6; Figure 2; *P* < 0.01).

### 3.2. Microscopical Analysis

Prior to the main study, three rats (number of burn wounds = 6) were sacrificed after the application of the burn stamp and wounds were excised and applied to histological assessment. All wounds revealed affection of the epidermal as well as the reticular dermal layer, which classified them as deep partial-thickness burns.

Histological analysis revealed no signs of infection.

**Figure 3:**
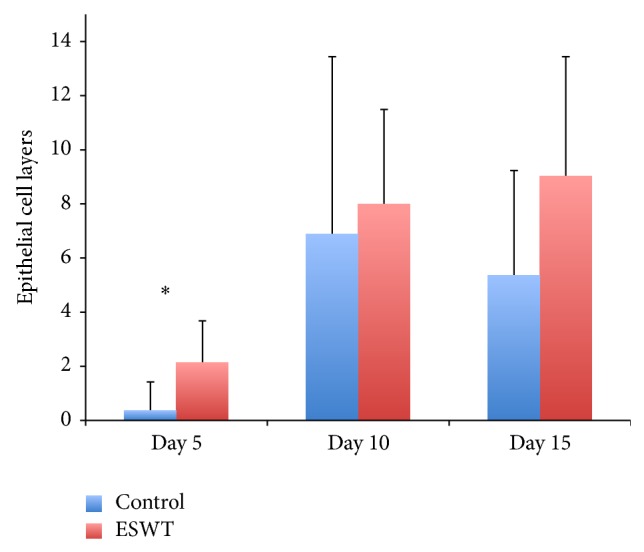
Number of epidermal cell layers at the centre of the burn wound of the control group compared to the ESWT-treated group. Day 5 shows statistical significance with a *P*-value of 0.038.

Counting of epithelial cell layers showed significant differences between the ESWT group (2.1 ± 1.6 layers) and the control group (0.4 ± 1.1 layers) on day 5 (Figure 3; *P* < 0.05). On day 10 (group 1: 8 ± 3.6 layers, group 2: 6.9 ± 6.6 layers) and day 15 (group 1: 9 ± 4.4 layers, group 2: 5.4 ± 3.5 layers), the ESWT group showed a greater number of cell layers than animals of the control group. However, this difference did not reach statistical significance.

**Figure 4:**
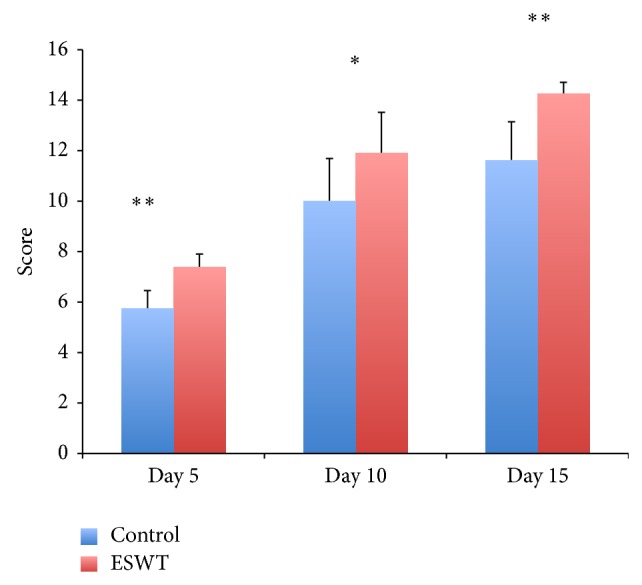
Calculated score after Schlager et al. [10] of burn wound healing in the control compared to the ESWT-treated group on days 5, 10, and 15 (^*^ *P* < 0,05, ^**^ *P* < 0,01).

The wound score system showed a highly significant improvement of the healing process on day 5 (group 1: 7.4 ± 0.5; group 2: 5.8 ± 0.7) and day 15 (group 1: 14.3 ± 0.5; group 2: 11.6 ± 1.5) and a significant improvement of the healing process on day 10 (group 1: 11.9 ± 1.6; group 2: 10.0 ± 1.7) (Figure 4).

**Figure 5:**
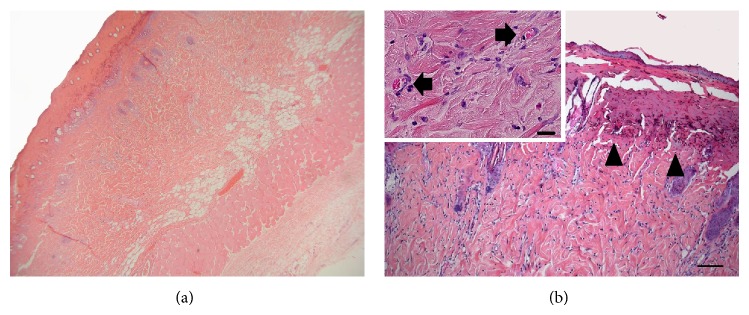
(a) This haematoxylin-eosin- (H.E.-) stained histological section shows the centre of the burn wound of the control group on day 5. (b) The burn wound has not yet reepithelialized (arrowheads); vessels in the reticular stratum are rare (scale bar = 100 *μ*m). The inset shows the distribution of vessels (arrows) in the reticular stratum in a higher magnification (scale bar = 20 *μ*m).

**Figure 6:**
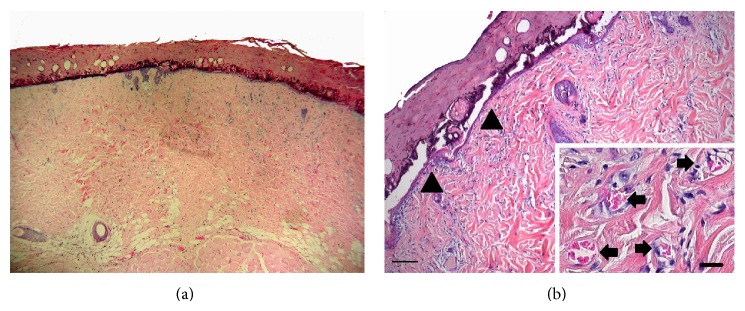
(a) The H.E.-stained histological section shows the centre of the burn wound of the ESWT-treated group on day 5. (b) The burn wound is closed by a thin continuous layer of epithelial cells (arrowheads). The reticular layer shows a higher number of vessels, compared to the control group (scale bar = 100 *μ*m). The inset shows the distribution of vessels (arrows) in the reticular stratum in a higher magnification (scale bar = 20 *μ*m).

Macroscopical and histological findings are summarized in Table 2. Wound surface decreased in both groups over time; however, just one animal of the ESWT group showed a minimally open wound ground on day 15. Moreover, the perfusion index showed a highly significant difference in perfusion on day 5. According to these findings, the ingrowth of new epithelial tissue into the wounds was faster and differed significantly on day 5 in the ESWT group. The wound score, which summarizes histological parameters like epithelialization, cellular content, granulation, and vascularity, differed significantly on all days of measurement and thus indicated the formation of a well vascularized and reepithelialized tissue. During the entire investigation period group 1 showed a higher reepithelialization rate in the wound center, more vessels in the reticular layer, and a faster-developing granulation, accompanied by a faster appearance of fibroblasts, compared to the untreated group.  Figure 6 shows the wound areas of the ESWT group and Figure 5 the control group, respectively. The wounds in the ESWT group already showed a thin but continuous epithelial covering. Vessels in the reticular stratum were common.

## 4. Discussion

Shock waves are high-amplitude sound waves that extend in a three-dimensional manner. They are characterized by a fast and intense pressure boost, in which a positive pressure phase is followed by a negative one [11].

ESWT has shown its effectiveness in urology, in orthopedics, and recently in plastic and reconstructive surgery [1–6, 12–16].

In the current study we found that the treatment with this noninvasive technique resulted in a clinical tendency towards better wound healing, shown by a significantly smaller and well-epithelialized wound area in the treatment group on day 15. Interestingly, on day 5 the measured wound area of the ESWT-treated group was still larger than the respective one of the untreated group. One possible explanation of this phenomenon is that this initially delayed wound closure could be due to an additional tissue injury caused by the highly energetic shock waves, as observed in bones [17] and endothelial cells [18, 19]. Petechial bleeding observed immediately after the shock-wave treatment lends some support to this assumption. However, in previous investigations performed by our group, no such bleeding was observed [3–5]. A possible explanation for this observation might be that in those studies shock waves were applied to uninjured skin whereas in the current study to skin that had undergone burn injury. It is fair to assume that injured skin is liable to be more sensitive to additional forces.

Already on day 5 a significantly higher number of epithelial cell layers could be counted in the burn injury center, although the macroscopically observed wound area was yet larger. This trend could be seen during the entire investigation period. Histological examination and the wound score evaluated important factors of wound healing (granulation, vascularization, epithelialization, and cellular content) [10]. During the entire investigation period, the ESWT group showed a higher reepithelialization rate in the wound center and more vessels in the reticular layer compared to the untreated group. Moreover, a lower number of inflammatory cells, less fibroblasts, and a uniform layer of granulation could be seen. Thus, a significantly better healing on days 5, 10, and 15 could be shown. These effects may be mediated by growth factors, which are known to be upregulated after ESWT and moreover within the first days after trauma and damage to the skin [14, 15, 20–23]. However, this assumption is highly speculative, due to the fact that staining of these factors was not performed in our study. With the establishment of a day 1 group of killed rats, the wound score system might have shown proper time comparison between the control and the treatment group. Nevertheless, we abstained from a further group to decrease the number of sacrificed rats needed for this study.

Inflammatory response and formation of granulation tissue, however, play a major role in burn wound healing. It is a well-known fact that rats heal mostly by contraction. Therefore, the histological findings of reepithelialization and the reduction of the number of fibroblasts and inflammatory cells in the ESWT group stay even more for an improvement in the healing of the burn wound after ESWT.

Wound perfusion was measured with the Laser Doppler Imager. The Laser Doppler perfusion image index (LDPII) is a standard tool in the determination of perfusion and microcirculation in the investigation of different diseases correlating to tissue ischemia [24, 25]. It directly correlates with arteriogenesis [26]. Blood flow in ESWT-treated rats significantly increased over the first five days. ESWT is known to contribute to the release of growth and angiogenic factors [4]. Therefore, together with the physiological response after trauma, ESWT may lead to an immediate increase of capillary perfusion and thus to a better blood supply and perfusion as well as to prevention of ischemia, a major problem in the zone of stasis of an acute burn wound. The increased number of vessels in the reticular layer of wounds of the ESWT group through all specimens supports these theories. Huemer et al. showed similar results after shock wave treatment of abdominal skin flaps [5]. An alternative working mechanism of ESWT could be vasodilatation and thus increased perfusion of the damaged tissue, as already shown in the literature [27].

## 5. Conclusion

The present study demonstrates significant benefits of ESWT in regeneration of skin and wound closure after deep partial-thickness burns. These findings might be confirmed by the fact that in first clinical reports deep partial-thickness burns show the tendency to heal without any surgical intervention when they are treated with ESWT [28]. We believe that ESWT represents a simple, noninvasive, and cost-effective treatment approach to deep partial-thickness burns, which may help avoid skin grafting or flap surgery by exploiting the vascular supply that is left intact after burn injuries and by accelerating the wound healing process. Nevertheless, further investigations, for example, a multiarm study with multiple ESWT treatments over a set treatment period instead of one treatment immediate status after injury, have to be performed to fully understand the mechanisms and benefits of ESWT treatment on burns.

## Figures and Tables

**Table 1:** Histologic scoring of burn wounds according to Schlager et al. [10].

1	EP	None to very minimal epithelialization
	CC	None to very minimal (mainly inflammatory cells)
	GT	None
	V	None

2	EP	Minimal epithelialization
	CC	Predominately inflammatory cells, few fibroblasts
	GT	None to a thin layer of granulation tissue
	V	Few capillaries

3	EP	Completely thin layer
	CC	More fibroblasts, still with inflammatory cells
	GT	Thicker layer of granulation tissue
	V	Well-defined capillary system

4	EP	Completely thick layer
	CC	Fewer numbers of fibroblasts in dermis
	GT	Uniformly thick layer of granulation tissue
	V	Extensive neovascularization

EP: epithelialization; CC: cellular content; GT: granulation tissue; V: vascularity.

**Table 2:** Summary of macroscopical and histological findings of the ESWT group compared to the control group.

Day	Group	Wound surface		Perfusion index		Epithelial cell layers		Score system	
		Mean	SD	Mean	SD	Mean	SD	Mean	SD
5	Control	43.6	8.6	92.7	32.6	0.4	1.1	5.8	0.7
	ESWT	48.5	10.0	145.2^**^	22.7	2.1^*^	1.6	7.4^**^	0.5

10	Control	7.9	8.8	86.0	21.7	6.9	6.6	10.0	1.7
	ESWT	6.8	6.6	83.3	16.9	8.0	3.6	11.9^*^	1.6

15	Control	3.8	5.5	59.5	20.9	5.4	3.9	11.6	1.5
	ESWT	0.07^*^	0.1	70.3	9.8	9.0	4.4	14.3^**^	0.5

^*^ *P* < 0.05; ^**^ *P* < 0.01.
